# Loss of the podocyte glucocorticoid receptor exacerbates proteinuria after injury

**DOI:** 10.1038/s41598-017-10490-z

**Published:** 2017-08-29

**Authors:** Han Zhou, Xuefei Tian, Alda Tufro, Gilbert Moeckel, Shuta Ishibe, Julie Goodwin

**Affiliations:** 1 Department of Pediatrics, Yale University School of Medicine, New Haven, CT 06520 USA; 2 Department of Internal Medicine, Yale University School of Medicine, New Haven, CT 06520 USA; 3 Department of Pathology, Yale University School of Medicine, New Haven, CT 06520 USA

## Abstract

Nephrotic syndrome is a common disorder in adults and children whose etiology is largely unknown. Glucocorticoids remain the mainstay of therapy in most cases, though their mechanism of action remains poorly understood. Emerging evidence suggests that immunomodulatory therapies used in nephrotic syndrome directly target the podocytes. To study how steroids directly affect the podocytes in the treatment of proteinuria, we created a mouse model with podocyte-specific deletion of the glucocorticoid receptor. The podocyte-specific glucocorticoid receptor (GR) knockout mice had similar renal function and protein excretion compared to wild type. However, after glomerular injury induced by either LPS or nephrotoxic serum, the podocyte GR knockout mice demonstrated worsened proteinuria compared to wild type. Ultrastructural examination of podocytes confirmed more robust foot process effacement in the knockout animals. Expression of several key slit diaphragm protein was down regulated in pGR KO mice. Primary podocytes isolated from wild type and podocyte GR knockout mice showed similar actin stress fiber staining patterns in unstimulated conditions. Yet, when exposed to LPS, GR knockout podocytes demonstrated fewer stress fibers and impaired migration compared to wild type podocytes. We conclude that the podocyte glucocorticoid receptor is important for limiting proteinuria in settings of podocyte injury.

## Introduction

Nephrotic syndrome is one of the most common kidney diseases in both children and adults. It is a multifactorial disease that manifests clinically with proteinuria, hypoalbuminemia, edema and hyperlipidemia, and is caused at the molecular level by a failure of the glomerular filtration barrier. Oral glucocorticoid therapy is the mainstay of treatment and induces remission in approximately 50% of adults and 80% of children^1, 2^. Complete or partial response to glucocorticoids is still considered the best prognostic factor in preserving long term renal function^3^. In addition, a significant subset of patients present with, or acquire, glucocorticoid resistance^4^, and others develop severe side effects rendering steroids intolerable^5^. Overall, the decision to treat with glucocorticoids remains largely empiric, since the mechanism of action whereby they induce nephrotic syndrome remission is not well understood and their target cells in this setting have not been clearly identified.

Over the past few decades, evidence has accumulated that the immune system plays an important role in triggering and/or maintaining nephrotic syndrome. Some of the earliest studies suggested a serum factor that decreased T cell function in patients with nephrotic syndrome^6, 7^. In subsequent work, the severity of nephrotic syndrome has been shown to be associated with decreased activity of regulatory T cells^8, 9^. B cells have recently also been shown to play a role in the pathophysiology of this condition through the therapeutic effect, in some patients, of rituximab, a B-cell inhibitor^10^. Studies in animal models have clearly shown a role for complement in the development of nephrotic syndrome^11, 12^ and nearly two dozen cytokines^13^, as well as the transcription factor NF-κB^14^, have also been implicated in the pathogenesis of this condition.

Podocyte foot process effacement is the ultrastructural hallmark of nephrotic syndrome, although it is often present in other renal diseases which are accompanied by nephrotic range proteinuria^15^. As such, the podocyte is considered to be the target of injury under these conditions^16^ and acquired podocytopathies are regarded mainly as immunological disorders^17^. The pathogenesis of nephrotic syndrome is often ascribed to dedifferentiation of podocytes leading to direct injury of the slit diaphragm and/or the actin cytoskeleton. Changes in the interaction between the glomerular basement membrane and the podocytes are likely to play a role^18^. With increasing knowledge of podocyte biology, these specialized cells are now widely regarded as direct target of immunosuppressive therapy^18, 19^.

It has been shown that glucocorticoid receptors are expressed in the human podocyte and translocate to the nucleus upon treatment with dexamethasone^20^. *In vitro* studies have demonstrated that glucocorticoids protect podocytes from injury by inducing actin filament stabilization^21^ and protect podocytes from apoptosis induced by puromycin aminonucleoside^22^. Of course the mechanistic link between administration of steroids, acting through cell-specific GR, and suppression of inflammation is central to this process.

In this study, we developed a novel mouse model with podocyte-specific deletion of the glucocorticoid receptor. Podocyte GR KO (pGR KO) mice showed no developmental phenotype, survived to adulthood and did not develop proteinuria in control conditions. However, podocyte GR KO mice demonstrated more severe renal injury than control littermates upon exposure to either a systemic insult or a renal-specific insult. We conclude that podocyte GR is essential to mitigate proteinuria after injury and propose that directly targeting podocyte GR may play a vital role in the treatment of nephrotic syndrome and other podocytopathies.

## Results

### Generation and characterization of podocyte-specific GR KO mice

**Figure 1:**
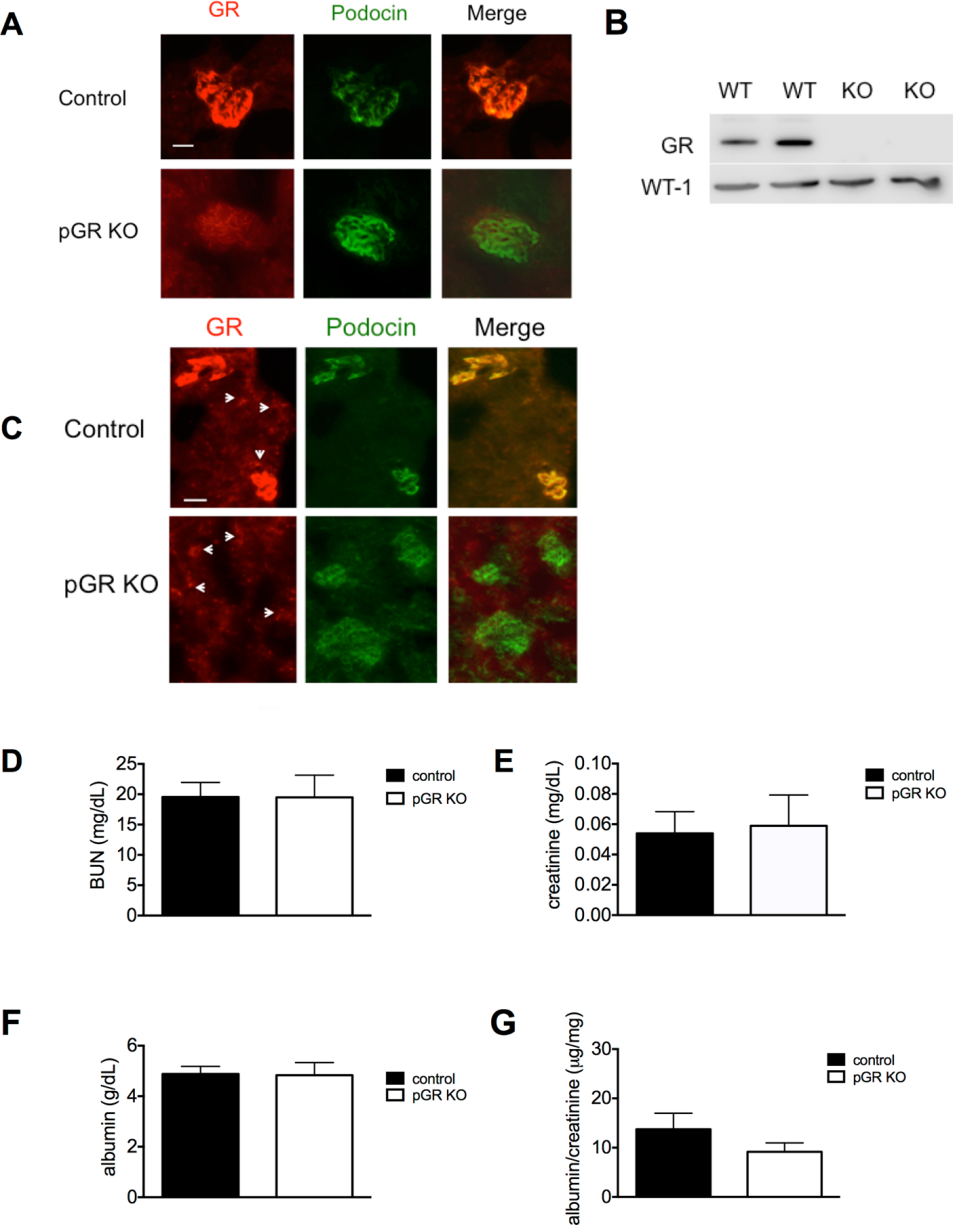
Effective KO of GR in podocytes. (**A**) Immunofluorescent staining of glomeruli with antibodies to GR and podocin showing co-localization in control mice (top panels) and efficient podocyte GR knockout (bottom panels). Scale bar 25 μm. (**B**) Western blot of primary podocytes isolated from mice of both genotypes showing GR deletion in pGR KO mice. WT-1 is shown as a podocyte loading control. (**C**) Lower power view of immunofluorescent staining demonstrating preservation of GR in tubular structures (arrowheads). Scale bar 50 μm. Phenotyping of pGR KO mice at baseline shows no difference in (**D**) BUN, (**E**) serum creatinine, (**F**) serum albumin or (**G**) urine albumin/creatinine ratios compared to Cre- littermate controls. n = 7 mice/group.

Mice carrying the floxed GR allele (*GR* ^*fl/fl*^)^23^ were crossed with mice expressing the *podocin*-*Cre* transgene^24^. The resulting *podocin*-*Cre*+*/GR* ^*fl/*+^ heterozygotes were crossed to produce the desired genotype, *podocin*-*Cre*+*/GR* ^*fl/fl*^, here identified as pGR KO. These mice were born at the expected Mendelian frequency indicating that podocyte GR deletion does not lead to embryonic lethality. Podocyte-specific GR (pGR) deletion was confirmed by immunocytochemistry of kidney sections (Fig. 1A) as well as by Western blot of isolated enriched primary podocytes (Fig. 1B). Colocalization of immunoreactive GR with podocin in control mice documented podocyte GR protein expression *in vivo* (Fig. 1A). Lower power views of immunofluorescent sections demonstrate preservation of GR in tubular structures in mice of both genotypes (Fig. 1C). No perinatal mortality was observed in pGR KO mice indicating that podocyte GR is not required for postnatal renal development. pGR KO mice gained weight normally and demonstrated normal activity and behavior until at least 1 year of age. The pGR KO mice failed to reveal an overt baseline renal phenotype as evidenced by similar serum albumin, BUN, and creatinine measurements and urine albumin/creatinine ratios as compared to *Cre-* *GR* ^*fl/fl*^ littermate controls (Fig. 1D–G).

### Podocyte GR KO worsens LPS-induced proteinuria

**Figure 2:**
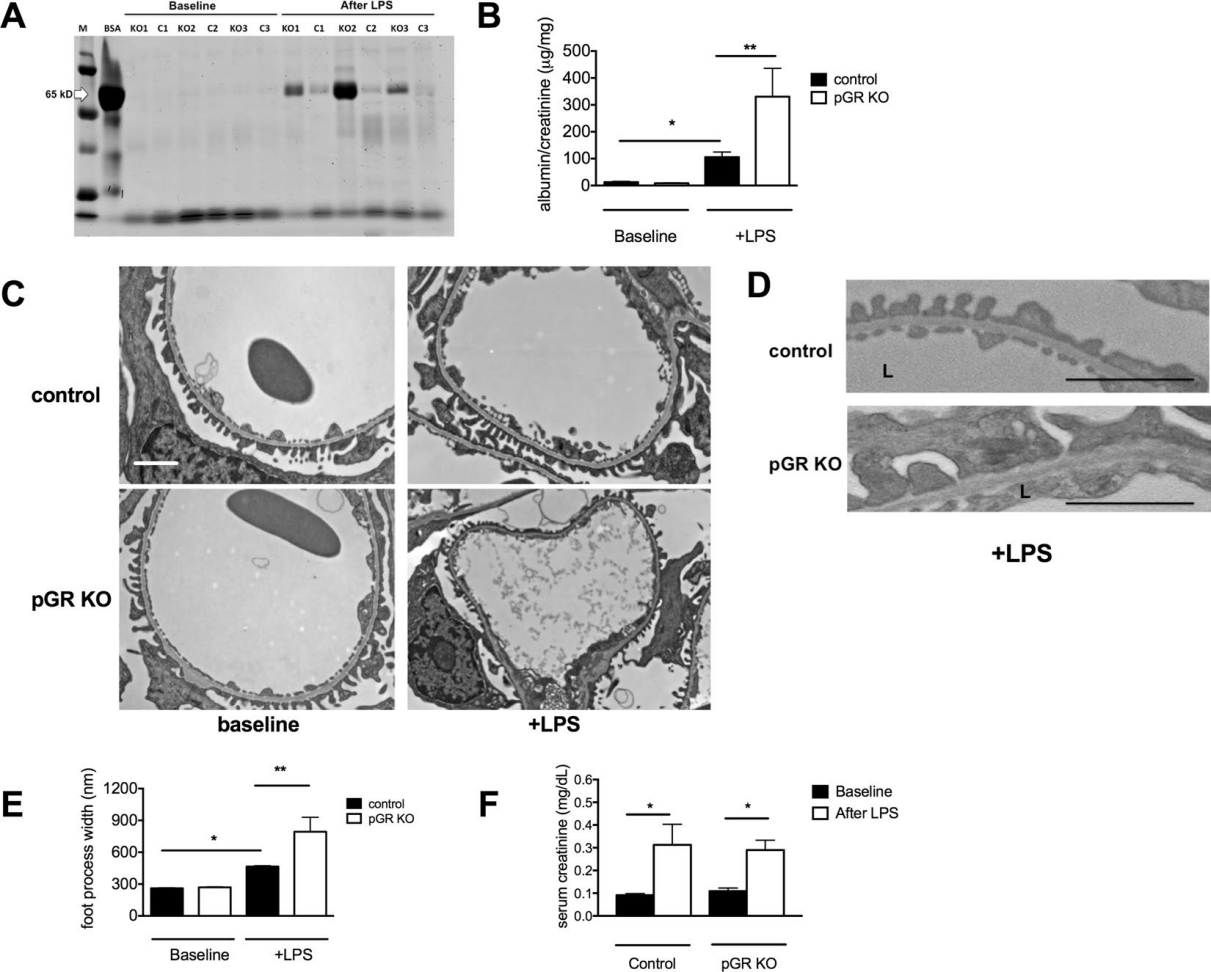
pGR KO mice demonstrate worsened LPS-induced proteinuria and foot process effacement. (**A**) Representative Coomassie blue stain of urine from control (C1-3) and pGR KO (KO1-3) mice before and after treatment with LPS, resolved by SDS-PAGE; BSA (5 μg) standard indicates albumin molecular weight. Equal urine volumes (5 μl) were loaded in each lane. (**B**) Quantification of urine albumin/creatinine ratios before and after exposure to LPS. (**C**) Representative TEM images from control and pGR KO mice before and after LPS. Images’ original magnification is 6000x. (**D**) Areas from C are shown at higher magnification and illustrate more severe podocyte injury in LPS-treated pGR KO mice than in control mice. Scale bar 1 μm. L denotes capillary lumen side of GBM. (**E**) Quantification of podocyte foot process width before and after LPS in control and pGR KO mice. (**F**) Serum creatinine before and after LPS. n = 7 mice/group *p < 0.05, **p < 0.01.

To examine the function of podocyte GR under stress conditions *in vivo*, we challenged control (*podocin*-*Cre*-*/GR* ^*fl/fl*^) and pGR KO mice with LPS, an agent that is well known to induce acute transient proteinuria in mice^25^. Twenty-four hours following low dose (12.5 mg/kg) intraperitoneal LPS injection, pGR KO mice developed more severe proteinuria than control mice, as demonstrated by Coomassie Blue staining (Fig. 2A) and validated by urine albumin/creatinine ratios (Fig. 2B). Renal function was found to be similarly impaired after LPS in pGR KO and control mice (Fig. 2F). To investigate whether the increased proteinuria was due to further damage of the glomerular filtration barrier, we examined the kidney ultrastructure by transmission electron microscopy (TEM) in LPS- and vehicle-treated pGR KO and control kidneys. As expected, there was no ultrastructural difference in vehicle-treated pGR KO and control kidneys. However, LPS-treated pGR KO mice demonstrated more severe podocyte foot process effacement than LPS-treated control mice (Fig. 2C–E). Quantification of foot process width revealed a nearly 2-fold larger mean foot process width change in LPS-treated pGR KO compared to LPS-treated control mice (Fig. 2E). Additional images of representative TEMs from mice of both genotypes after LPS treatment are shown in Supplementary Figure 1.

To assess whether possible differences in systemic inflammation existed between control and pGR KO mice, we examined serum IL-6 and Tissue Factor (F3) mRNA expression 24 hours after LPS injection. We observed no differences in the expression of either gene (Supplementary Figure 2) suggesting the degree of systemic inflammation was similar between the control and pGR KO mice and therefore could not account for the worsened proteinuria and foot process effacement observed in the pGR KO mice.

### Podocyte GR KO exacerbates nephrotoxic serum (NTS)-induced proteinuria

To test the role of podocyte GR in response to renal-specific injury, we used the nephrotoxic serum (NTS) model of crescentic glomerulonephritis which is well characterized^26^. Briefly, mice were immunized with rabbit IgG. Six days later glomuerulonephritis was induced with injections of rabbit anti-mouse GBM serum on days 1, 2 and 3 as described^27^. Similar deposition of anti-rabbit IgG in the glomerular basement membrane was verified in mice of both genotypes (Supplementary Figure 3).

**Figure 3:**
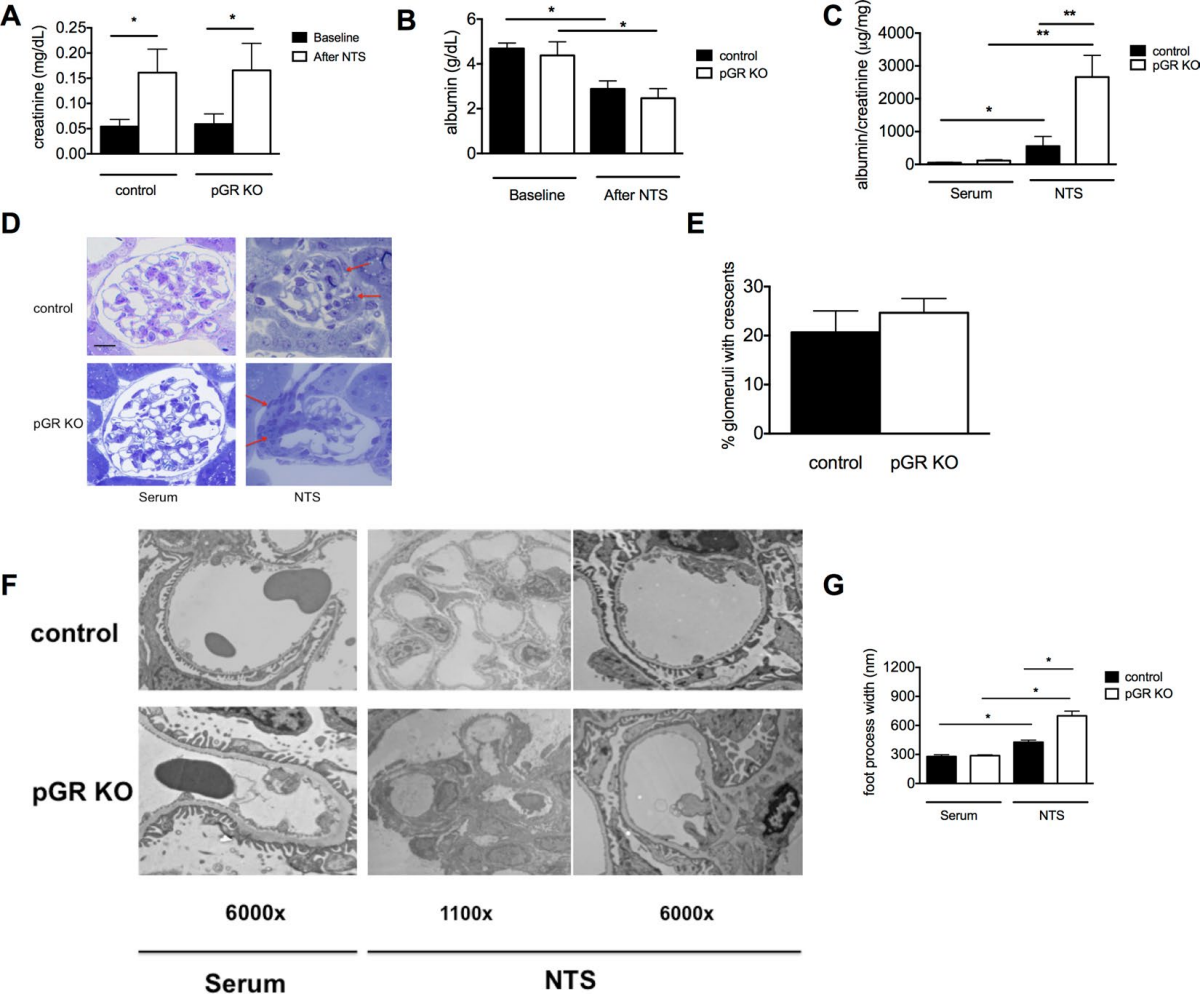
NTS induces crescentic GN and nephrotic syndrome with more severe proteinuria in pGR KO mice. (**A**) Serum creatinine before and 1 week after NTS. (**B**) Serum albumin before and 1 week after NTS. (**C**) Quantification of urine albumin/creatinine ratios in serum-treated and NTS-treated mice of both genotypes shows ~4-fold increase in pGR KO mice. (**D**) Representative Toluidine blue stained images of serum-treated and NTS-treated mice of both genotypes. Red arrows indicate cellular crescents. Images shown at 40x. Scale bar 10 μm. (**E**) Percentage of glomeruli developing crescents after NTS treatment. (**F**) Representative TEM images of serum-treated and NTS-treated mice. Note the severely disorganized architecture that can be appreciated at low power in the NTS-treated pGR KO glomerulus, and complete foot process effacement at higher power. (**G**) Quantification of podocyte foot process width. n = 8 mice/group *p < 0.05, **p < 0.01.

One week after the administration of the final NTS dose, spot urine samples were collected, and mice were sacrificed for blood and tissue processing. Following NTS, both control and pGR KO mice developed severe acute kidney injury (AKI) and nephrotic syndrome, as evidenced by approximately a three-fold increase in serum creatinine (Fig. 3A), and hypoalbuminemia (Fig. 3B), respectively. However, pGR KO mice demonstrated a significantly higher level of proteinuria than Cre- control littermates (Fig. 3C).

To assess the severity of NTS-induced renal injury in both genotypes, we performed quantitative histology and TEM. As expected, histology revealed crescentic glomerulonephritis in both control and pGR KO mice (Fig. 3D).

The percentage of crescentic glomeruli induced by NTS was similar in control and pGR KO mice (Fig. 3E). Representative sections are shown in Fig. 3F. TEM demonstrated extensive foot process effacement in both NTS-treated genotypes as compared to normal serum-treated mice. Importantly, quantitation of foot process width demonstrated that NTS-treated pGR KO mice have significantly wider foot processes than control mice (Fig. 3G), consistent with their more severe proteinuria.

### Response to protamine sulfate infusion and heparin rescue

We also examined the podocyte morphology in response to protamine sulfate infusion in mice of both genotypes. This form of renal injury is mediated by alterations in the charge of the GBM, is reversible with heparin infusion and thus not transcriptionally mediated. We found a strong trend toward worsened foot process effacement, though not reaching statistical significance, in the pGR KO mice treated with both protamine sulfate and with heparin rescue compared to the control mice under similar conditions. There was a small, but statistically significant difference between the foot process widths of pGR mice treated with vehicle and those treated with protamine sulfate and heparin (177 ± 13.6 nm vs. 277 ± 12.79 nm, p = 0.03) which was not found in the control animals under the same conditions (179.3 ± 8.9 nm vs. 228 ± 9.3 nm, p = 0.51) (Supplementary Figure 4).

### Examination of slit diaphragm protein expression

**Figure 4:**
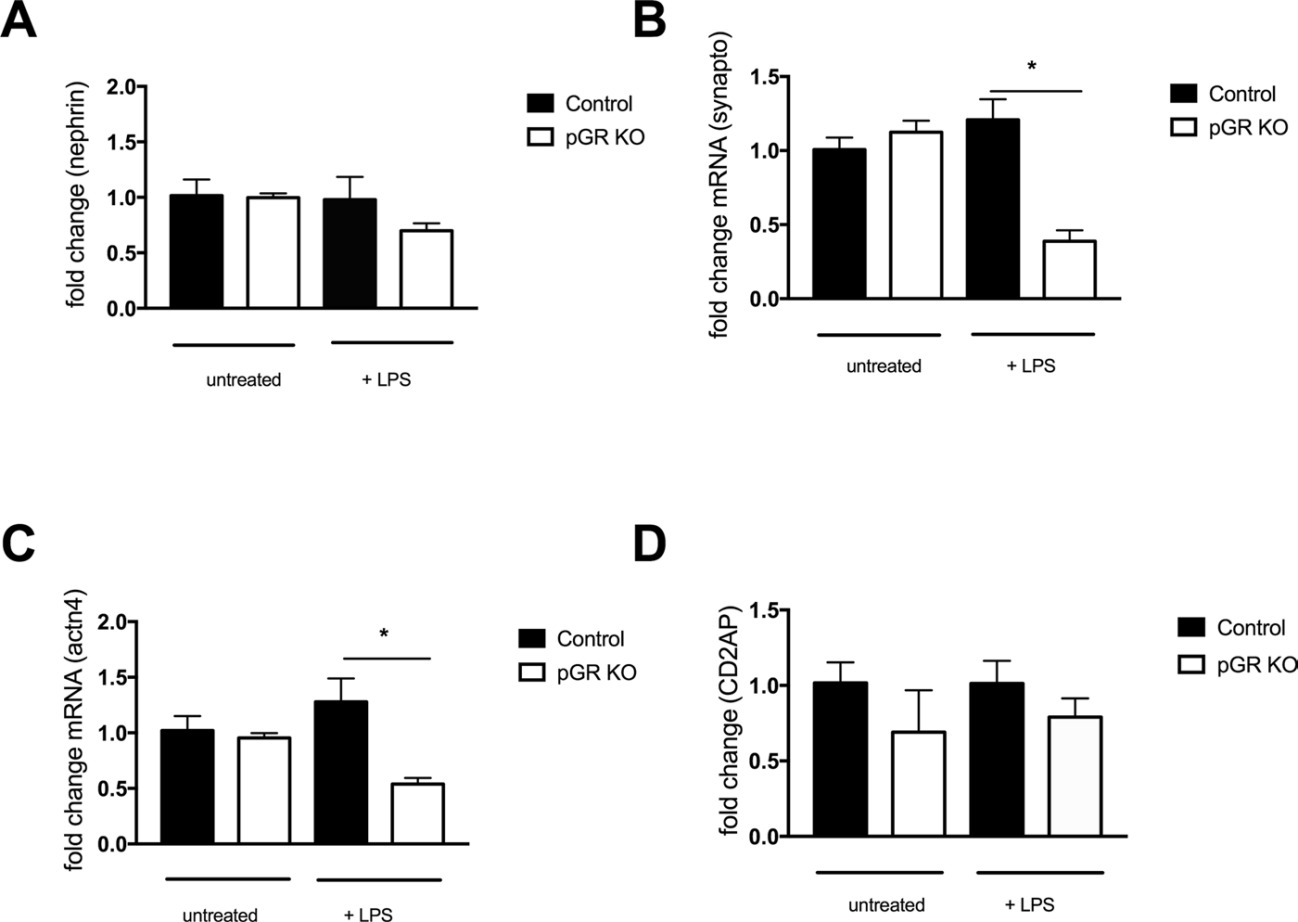
Slit diaphragm expression in untreated mice (n = 3/genotype) and mice treated with LPS (n = 5/group). qPCR for (**A**) nephrin, (**B**) synaptopodin, (**C**) alpha actinin 4 and (**D**) CD2AP was performed in control and pGR KO mice treated with LPS. Fold change was calculated for all groups and normalized to the untreated control group. *p < 0.05.

**Figure 5:**
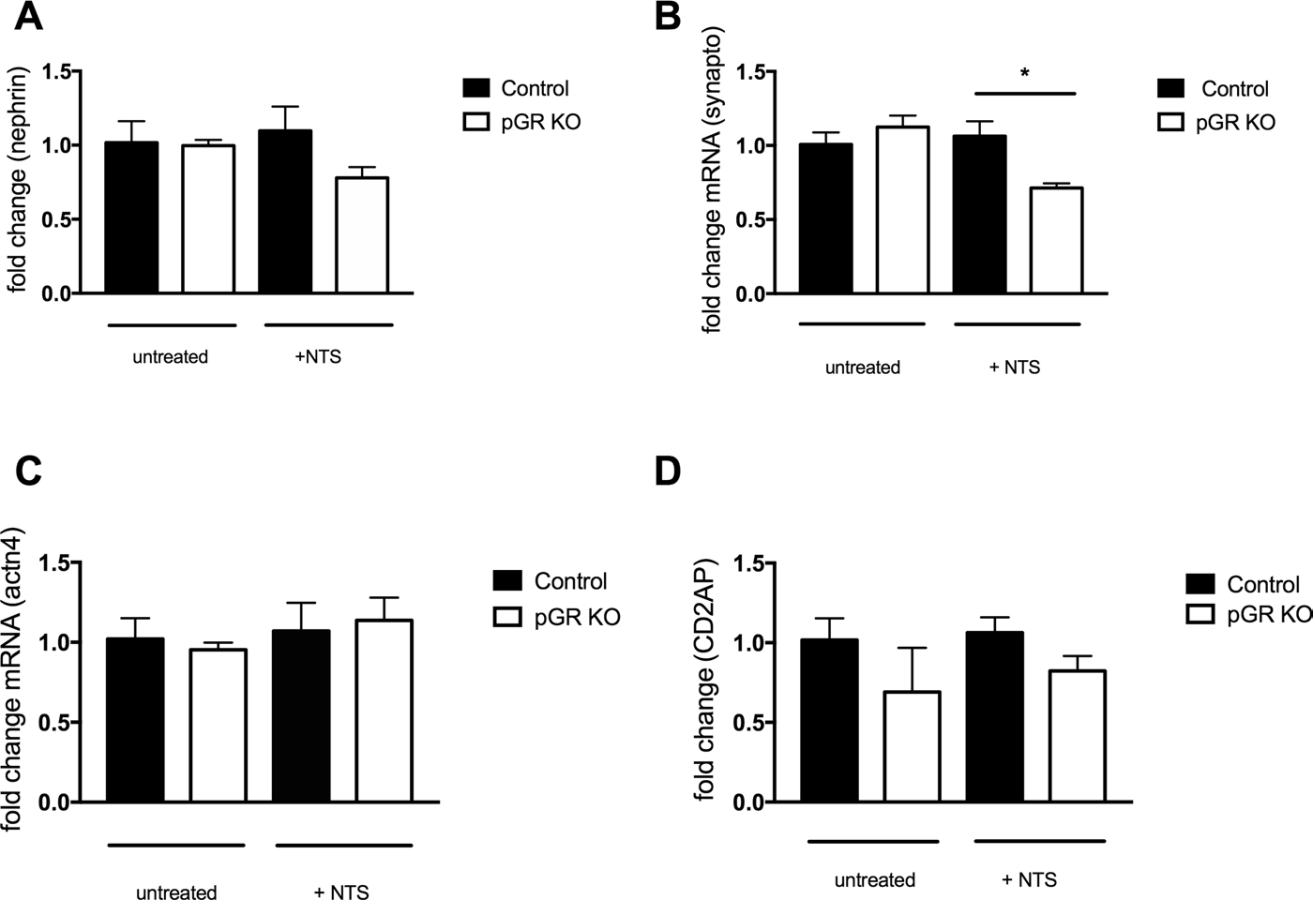
Slit diaphragm expression in untreated mice (n = 3/genotype) and mice treated with NTS (n = 5/genotype). qPCR for (**A**) nephrin, (**B**) synaptopodin, (**C**) alpha actinin 4 and (**D**) CD2AP was performed in control and pGR KO mice treated with NTS. Fold change was calculated for all groups and normalized to the untreated control group. *p < 0.05.

To begin to understand the mechanism underlying the enhanced proteinuria and foot process effacement observed in pGR KO mice subjected to these injury models, we examined the mRNA expression of key podocyte proteins: nephrin, synaptopodin, alpha-actinin 4 and CD2AP. Upon exposure to LPS, pGR KO mice showed a significant decrease in the expression of synaptopodin and alpha-actinin 4 compared to control littermates; there was also a trend towards decreased CD2AP and nephrin expression (Fig. 4). When subjected to the NTS model, synaptopodin decreased significantly in pGR KO mice, while nephrin and CD2AP mRNAs decreased mildly, not reaching statistical significance (Fig. 5).

### Podocyte GR KO alters F-actin stress fiber pattern after injury

**Figure 6:**
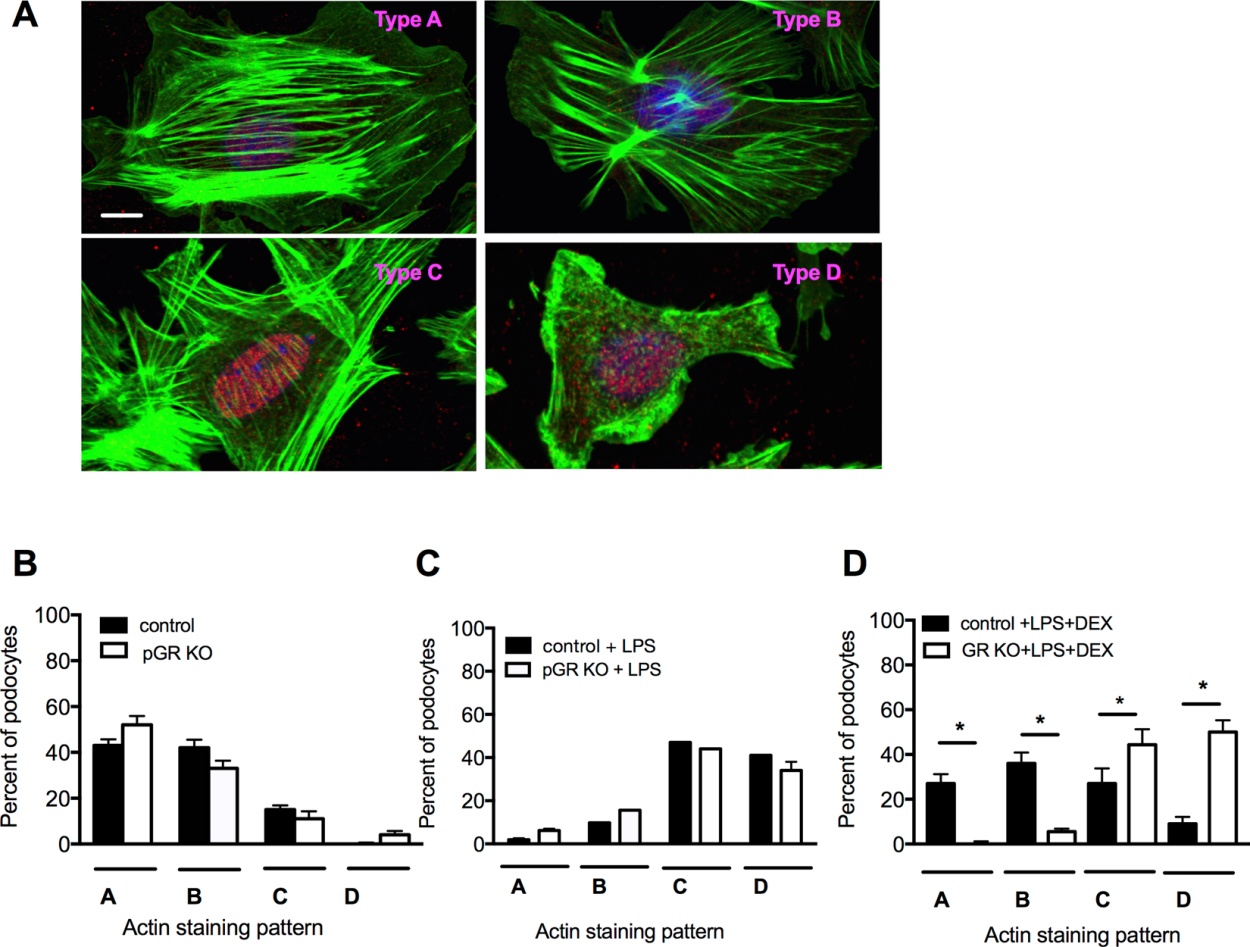
Actin stress fibers are reduced in GR KO podocytes after LPS. (**A**) Representative images of different phalloidin staining patterns in podocytes. Green-phalloidin, red-WT-1, blue-DAPI. Scale bar 10 μm. (**B**) Quantification of baseline F-actin staining patterns in control and GR KO podocytes. (**C**) Quantification of F-actin staining patterns in control and GR KO podocytes treated with LPS alone. (**D**) Quantification of F-actin staining patterns in control and GR KO podocytes treated with LPS + DEX. n = 3 separate experiments with at least 50 cells/experimental condition evaluated, *p < 0.05.

F-actin is the backbone and one of the critical structures of podocyte foot processes^28^. Therefore, to investigate how loss of pGR led to worsened foot process effacement, in the LPS injury model, we evaluated F-actin stress fiber patterns in enriched primary podocytes isolated from pGR and control littermates. Podocytes were subjected to phalloidin staining, imaged and classified into one of 4 staining patterns as previously described^29^ (Fig. 6A). When unstimulated, control and pGR KO podocytes had similar F-actin staining patterns (Fig. 6B), consistent with the *in vivo* observation that pGR KO mice have no proteinuria under control conditions. When stimulated with 10 μg/ml LPS for 4 hours, control and pGR KO podocytes showed similar injured actin stress fiber patterns, with a predominance of C and D patterns (Fig. 6C).

To determine the ability of steroids to rescue this injured F-actin phenotype we again treated cells with LPS and then exposed them to 100 nM dexamethasone (DEX) for 1 hour. Treatment with DEX clearly induced a shift in the F-actin stress fiber pattern back to the baseline A and B patterns in control podocytes effectively rescuing the phenotype. In contrast, DEX did not rescue the F-actin fibers of GR KO podocytes (Fig. 6D), as indicated by the ~10% vs. ~50% D fibers in control and GR KO podocytes, respectively. These results indicate that absence of the glucocorticoid receptor prevents injured podocytes from re-establishing a healthy F-actin fiber pattern, suggesting that restoration of the actin cytoskeleton after injury is GR-dependent.

### Migration defect of GR KO podocytes

**Figure 7:**
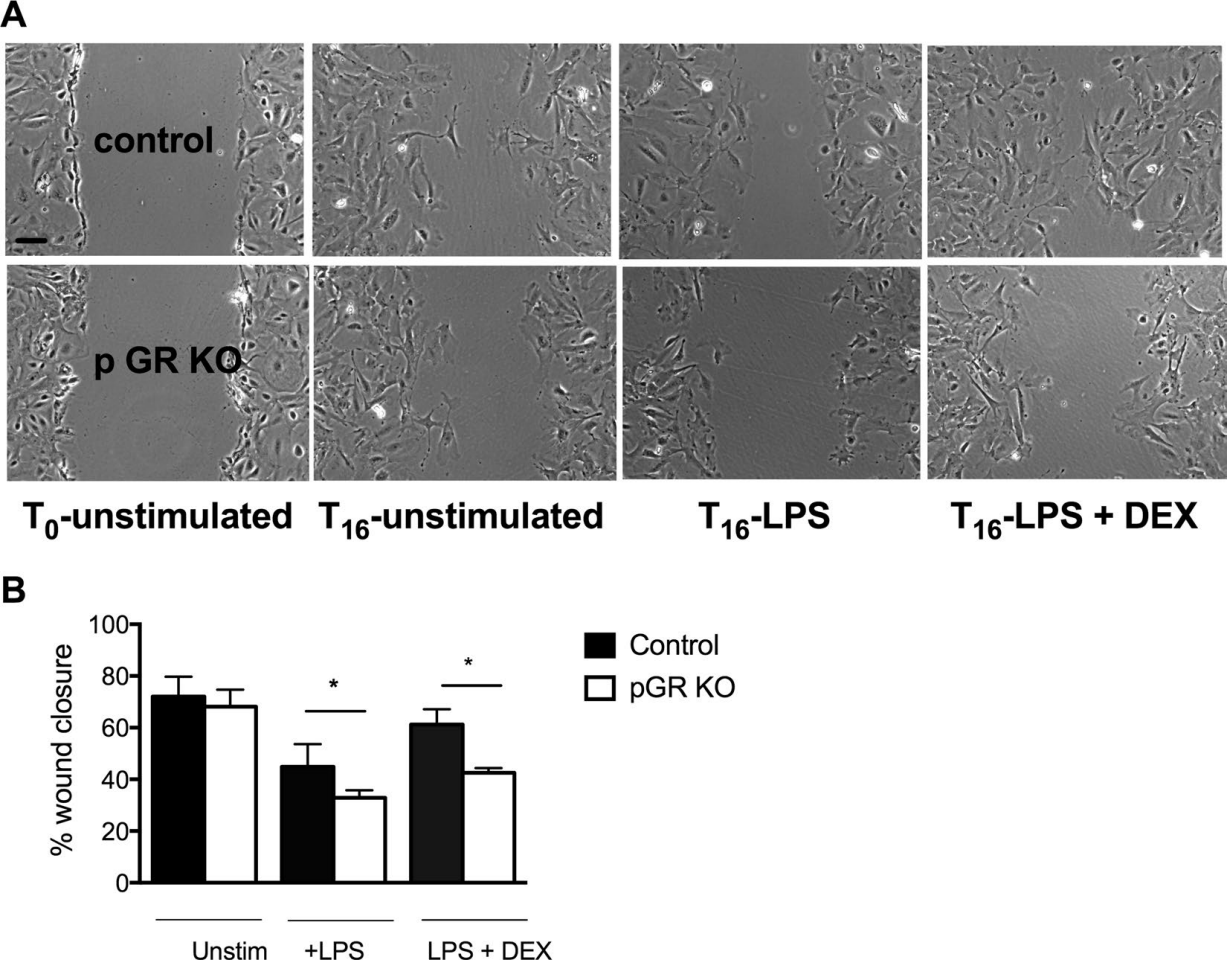
Impaired wound healing in GR KO podocytes after LPS. (**A**) Representative images of control and GR KO podocytes at time 0 following wound initiation, 16 hours after unstimulated wound healing, 16 hours after treatment with LPS 10 μg/ml and 16 hours after LPS 10 μg/ml + DEX 100 nM. Images shown at 20x magnification. Scale bar 25 μm. (**B**) Quantification of wound healing assay. n = 3 experiments, *p < 0.05.

To evaluate whether the observed difference in F-actin stress fiber patterns between control and GR KO podocytes results in changes in podocyte motility, we performed wound-healing assays. Confluent podocyte monolayers were exposed to LPS or vehicle with or without DEX. Under control conditions, control and GR KO podocytes migrated similarly, whereas, upon exposure to LPS, GR KO podocytes demonstrated reduced migration as compared to control podocytes (Fig. 7A). Furthermore, DEX treatment clearly rescued the migration phenotype induced by LPS in control podocytes (72 ± 7.7% vs. 61 ± 5.9%, p = NS), but did not rescue the GR KO podocyte migration defect (68 ± 6.5% vs. 43 ± 1.8%, p = 0.01) (Fig. 7A and quantified in Fig. 7B).

### Decreased pFAK expression GR KO podocytes

**Figure 8:**
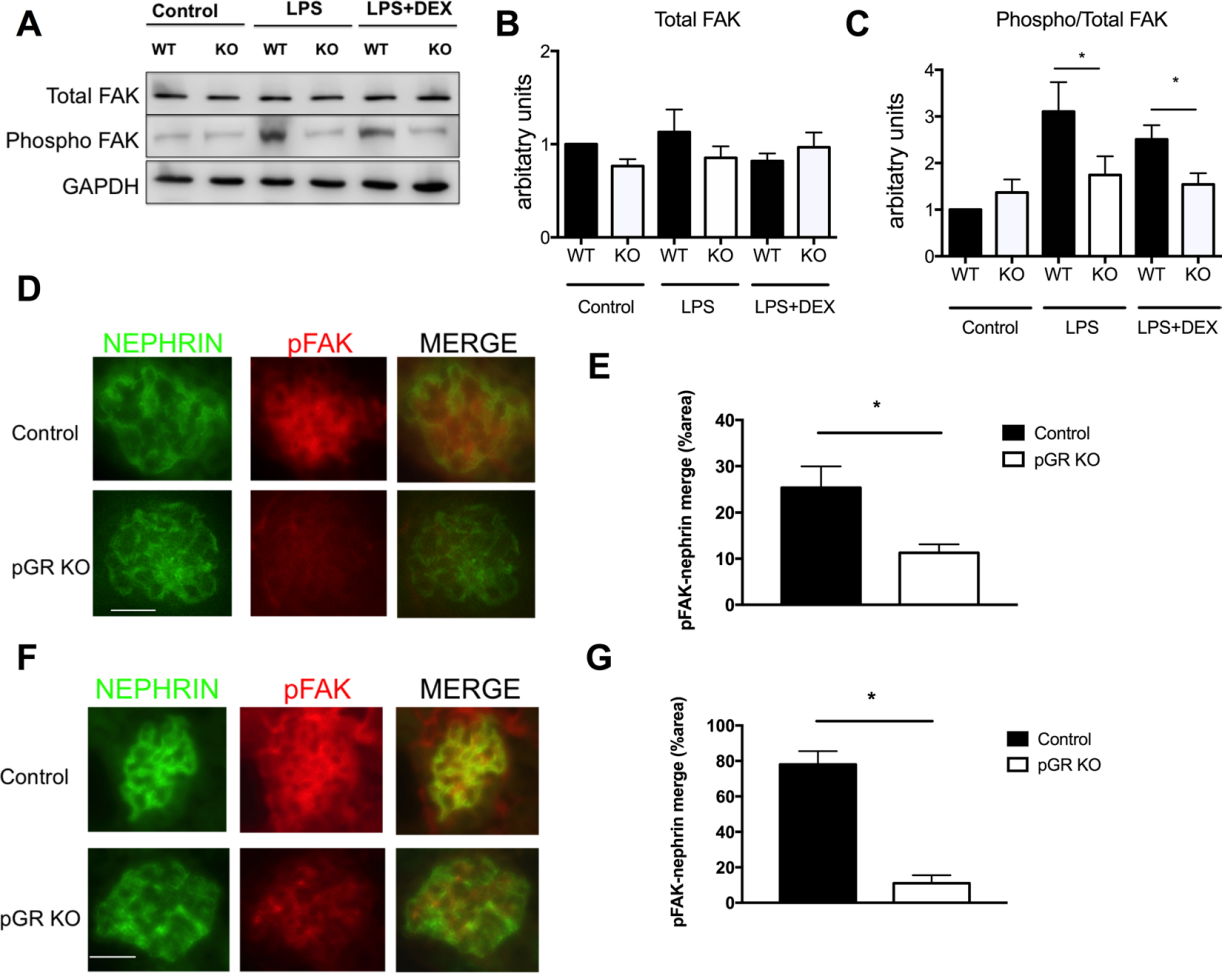
pFAK expression is increased in control but not GR KO podocytes after injury. (**A**) Representative Western blot of control and GR KO podocytes showing expression of total FAK and phospho-FAK under untreated, LPS-treated or LPS + DEX-treated conditions. Quantification of (**B**) total FAK expression normalized to GAPDH and (**C**) phospho-FAK expression normalized to total FAK. n = 3 experiments, *p < 0.05. (**D**) Representative images of glomeruli from LPS-treated control and pGR KO mice. (**E**) Quantification of glomerular pFAK/nephrin overlap in the LPS model. (**F**) Representative images of glomeruli from NTS-treated control and pGR KO mice. (**G**) Quantification of glomerular pFAK/nephrin overlap with the NTS model. Images shown at 40x, scale bar 25 μm.

To further investigate the migration defect observed in the GR KO podocytes, we examined the expression of focal adhesion kinase (FAK), a regulator of cell (and podocyte) migration, by immunoblotting and immunofluorescence. Control and GR KO podocytes were either unstimulated, exposed to LPS for 4 hours or exposed to LPS followed by 1 hour of DEX treatment. At baseline, total and phosphorylated FAK (pFAK, Tyr397) expression was similar in control and GR KO podocytes. Upon LPS exposure, control podocytes increased expression of pFAK as previously reported^27^, while pFAK expression decreased following administration of DEX as expected^30^. Quite surprisingly, in GR KO podocytes, pFAK remained unchanged after LPS exposure and DEX treatment despite the clearly worsened phenotype of these cells. Total FAK was similar in control and GR KO podocytes under all conditions (Fig. 8A–C). Lastly, pFAK expression was also evaluated by double immunofluorescence in glomeruli from control and pGR KO mice treated with either LPS or NTS. In both injury models we observed that podocyte pFAK expression was very low in pGR KO mice compared to control mice (Fig. 8D–G). Together, these findings suggest that GR is required for FAK phosphorylation in podocytes.

## Discussion

The major finding of this study is that loss of the podocyte glucocorticoid receptor worsens proteinuria following glomerular filtration barrier injury. Mechanistically we show that this occurs, in part, by impaired migration of injured podocytes lacking the glucocorticoid receptor, which are unable to activate FAK. There may be multiple explanations which could explain our observations including the possibilities that (i) GR plays a pleiotropic role in podocytes, (ii) GR may not be required for initial podocyte injury response but only for repair, (iii) GR may not be necessary for podocyte morphogenesis or (iv) there is a permissive role for endogenous steroids acting through podocyte GR. At this time we cannot fully explain our observations.

The role of steroids in the treatment of many renal conditions, including nephrotic syndrome, is well established, but the mechanisms by which they act are far from clear. Increasing evidence suggests that tissue-specific effects of GR can produce profound whole organism phenotypes^23, 31–33^. This line of reasoning is slowly extending to include renal diseases as well^34^. For example, two recent studies demonstrated the benefit of targeted drug delivery to the renal microvascular endothelial cells in a mouse model of anti-GBM glomerulonephritis^35, 36^. Another study by Morimoto *et al*. showed that cationic liposomes containing prednisolone phosphate could be preferentially targeted to mesangial cells and used to treat experimental glomerulonephritis in rats^37^. Lin *et al*. has demonstrated that 2-glucosamine can be used as a potential ligand for kidney-targeted delivery of dexamethasone in the treatment of ischemia-reperfusion injury^38^.

Previous work has demonstrated that dexamethasone can stabilize the expression of α-actinin 4^39, 40^ and increase the phosphorylation of nephrin in cultured podocytes^41^. Most recently, α-actinin 4 has been shown to directly interact with GR in human podocytes and act as a transcriptional regulator for steroid-modulated genes^42^. Dexamethasone has also been shown to down-regulate VEGF and inflammatory cytokines, including IL-6, in culture^43^. CD2AP, Nck and synaptopodin are known to be key slit diaphragm proteins^44, 45^, and it has also recently been shown that dexamethasone inhibits podocyte apoptosis by restoring CD2AP-PI3K-Akt signaling which is lost during cell injury by puromycin^45^. Additionally, dexamethasone has been demonstrated to induce expression of Krüppel-like factor 15, a zinc-finger transcription factor known to stabilize the actin cytoskeleton under conditions of cell stress, in both murine and human podocytes^46^. In this study we demonstrate significantly decreased expression of synaptopodin in pGR KO mice after NTS exposure as well as decreased alpha-actinin 4 and synaptopodin expression in pGR KO mice after LPS.

Though it is difficult to find a mouse model of nephrotic syndrome that closely replicates human disease, both the LPS model and the NTS model of crescentic glomerulonephritis are recognized as valuable tools for the study of nephrotic syndrome and proteinuric renal disease^47^. The NTS model is deemed superior by some for the study of the role of the podocyte in crescent formation and glomerular injury because Bowman’s capsule remains intact and no other infiltrating cells are required to mediate this injury^47, 48^. Here we report for the first time that the podocyte GR plays a protective role in the glomerular filtration barrier *in vivo* in the setting of acute systemic injury (LPS) and in crescentic glomerulonephritis (NTS), as evidenced by worsening proteinuria and podocyte injury in pGR KO mice. Of note the levels of proteinuria induced by both LPS and NTS in control animals in our study were similar to prior studies^29, 39^, further validating our results. The abnormal F-actin stress fiber pattern, podocyte-specific gene expression, and impaired podocyte migration observed in injured GR KO podocytes collectively indicate that GR plays a protective role in podocytes, consistent with the pGR KO *in vivo* findings.

Interest in renal GR physiology is growing. Agrawal *et al*. have recently shown that glucocorticoids can increase synaptopodin expression *in vivo* in a rat model of puromycin-aminonucleoside-induced proteinuria^49^. Kuppe *et al*. have highlighted the importance of GR in renal parietal epithelial cells in the development of crescentic glomerulonephritis using a model in which GR was knocked out from all renal epithelial cells by *Pax8*-*Cre* mediated recombination^50^. In contrast, we examined the specific role of GR solely in podocytes by inducing *Podocin*-*Cre* mediated recombination. Notably, GR deletion in neither all renal epithelial cells nor exclusively podocytes proved to cause a developmental or adult renal phenotype at baseline, proving that GR is dispensable for renal development and in normal physiological conditions. However, *pGR* KO and *Pax8*-*Cre* GR KO mice appear to differ in that the crescentic glomerulonephritis induced by NTS is clearly worsened in *pGR* KO and attenuated in *Pax8*-*Cre* GR KO mice. This discrepancy may be related to GR knock down in parietal epithelial cells in *Pax8*-*Cre* GR KO mice.

The finding that pFAK expression remained virtually unchanged in pGR KO cells after LPS treatment was quite unexpected. Since FAK activation has been shown to result in proteinuria and foot process effacement^27, 51, 52^, we had predicted a large increase in pFAK in the GR KO podocytes, which was neither observed *in vitro* nor *in vivo*. These results suggest that the podocyte glucocorticoid receptor is *required* for FAK activation. This is consistent with our finding of impaired migration in GR KO podocytes and previous reports showing that steroids reduce expression of pFAK in injured podocytes^30, 53^, as well as in other cell types^54, 55^. Additional evidence supports the idea that actin cytoskeletal integrity is required for DEX to promote the phosphorylation of FAK^56^. Though not demonstrated in podocytes, Koukouritaki *et al*. showed that rapid non-genomic effects of DEX on actin assembly and phosphorylation of FAK are co-dependent^56^. Therefore, GR deletion could potentially prevent FAK phosphorylation by eliminating the receptor through which both endogenous steroid and the synthetic ligand DEX would act. This observation may provide some explanation for the small but significantly worsened foot process effacement that we observed after protamine sulfate and heparin rescue in the pGR KO mice, since in this model with rapid reversal of renal injury, we would predict a mechanism related to non-genomic steroid effects and not transcriptional activation of pGR. Further studies will be required to investigate this phenomenon.

In conclusion, our study shows that loss of podocyte GR worsens proteinuria in settings of injury, both *in vivo* and *in vitro*. Since there was no detectable phenotype at baseline in the pGR KO mice we further conclude that GR acts as an important modifier to maintain the integrity of the filtration barrier under conditions of both systemic and renal-specific insult. Moreover, we show that GR KO renders podocytes unable to phosphorylate FAK, supporting the notion that pGR is a critical modulator of the actin cytoskeleton on multiple levels. Given the myriad side effects of systemic steroid use, identification of a downstream mechanism whereby podocyte-specific GR is able to regulate the glomerular filtration barrier would potentially allow targeted delivery of steroids directly to podocytes, which could have major therapeutic potential for proteinuric renal disease.

## Methods

### Generation of podocyte-specific glucocorticoid receptor KO mice

Female BL6 mice with floxed glucocorticoid receptor alleles, designated *GR* ^*loxP/loxP*^ ^57^, were mated with males possessing *Podocin*-*Cre* ^24^. By selective breeding, Podocin-Cre+/GR ^loxP/loxP^ homozygotes and Podocin-Cre-/GR ^loxP/loxP^ littermate controls were generated. These mice have been backcrossed for >12 generations and are congenic on a C57BL/6 background. Mouse DNA was isolated from tail clipping by standard methods and analyzed by PCR to determine mouse genotype. Primers used to detect the floxed glucocorticoid receptor allele were:

forward 5′ GGCATGCACATTACTGGCCTTCT 3′ and reverse 5′ CCTTCTCATTCCA TGTCAGCATGT 3′. Primers for detection of Cre were as follows: forward 5′ CCGGGCTGCCACGACCAA 3′ and reverse 5′ GGCGCGGCAACACCATTTTT 3′. All experiments were performed according to a protocol approved by the Institutional Animal Care and Use Committee at the Yale University School of Medicine and were in accordance with the National Institute of Health (NIH) Guidelines for the Care of Laboratory Animals.

### Antibodies and reagents

Antibodies used in this study were rabbit anti-WT1 (Santa Cruz Biotechnology, Inc), rabbit anti-GR (Santa Cruz Biotechnology, Inc.), goat anti-nephrin (Santa Cruz Biotechnology, Inc.), and rabbit anti-podocin (Sigma). Anti-phospho FAK (Tyr 397) (EMD Millipore), anti-FAK, clone 77 (Fisher Scientific) and GAPDH (Affinity Bioreagents) were also used. Complete Freund’s adjuvant was purchased from Sigma. Rabbit IgG was purchased from Jackson Immunoresearch Laboratories. Alexa Fluor 594 goat anti-rabbit IgG Ab and Alexa Fluor 488–conjugated phalloidin were purchased from Invitrogen. LPS serotype O55:B5 was purchased from Calbiochem and dexamethasone phosphate was purchased from MP Biomedicals.

### Serum and urine assays

Spot urine samples were obtained between 8–10 AM. Urine albumin and urine creatinine were measured by the George M. O’Brien Kidney Center Physiology Core at Yale University School of Medicine. Blood samples were obtained by retro-orbital puncture. Serum creatinine was measured by liquid-chromatography/tandem mass spectrometry. Urine creatinine was measured by the Quantichrome Creatinine Assay Kit (BioAssays Systems, California). Urine albumin was measured by the Mouse Albumin ELISA Quantitation Set (Bethyl Laboratories, Inc).

### Kidney histology, immunofluorescence and transmission electron microscopy

For histology, mice were anesthetized and perfused with 4% paraformaldehyde. Kidney sections were stained with Toluidine blue. For examination of the number of crescents present in NTS-treated animals, at least 50 glomeruli were examined from each mouse.

For immunofluorescence, frozen sections were stained as previously described^23^. Briefly, sections were fixed in 100% EtOH followed by rehydration in PBS. They were then permeabilized with 0.2% Triton and blocked with a solution of 10% serum and 1% BSA. Primary antibodies (anti-GR, anti-podocin, anti-nephrin and anti-FAK) were incubated overnight. Sections were washed and secondary antibodies were incubated for 45 minutes in the dark. Sections were washed again and coverslips were mounted using Vectashield. Sections were analyzed and quantified by ImageJ.

For electron microscopy studies, mice were anesthetized and perfusion fixed with 4% PFA and kidneys were isolated and immersion fixed in Karnovsky fixative for 2–3 hours. They were sectioned, mounted and imaged using a Nikon TE 2000U electron microscope. EM was performed by the Cellular and Molecular Physiology Core at Yale University. Image J was used for quantitative analysis of electron micrographs. Foot processes were measured from at least 50 μm of GBM for each mouse. Images were blind-coded with integer numbers prior to evaluation by someone other than the scorer.

### Nephrotoxic serum-induced podocyte injury

Rabbit anti-mouse GBM antibody (nephrotoxic serum) was generated by Lampire Biologic Laboratories and NTS-induced injury was performed as previously described^27^. Briefly, 8–12 week old mice were immunized with an IP injection of 200 μg of rabbit IgG in 200 μg of a 1:1 emulsion with complete Freund’s adjuvant. One week later (day 0) glomerulonephritis was induced with IV injections of 1:3 dilution of rabbit anti-mouse GBM serum for 3 consecutive days. Serum creatinine and urine albumin-to-creatinine ratios were measured on day 7. Pre-immune rabbit serum was used as a negative control.

### Isolation of primary podocytes

Podocytes from mice of both genotypes (*podocin Cre*- *GR* ^*fl/fl*^ and *podocin Cre* + *GR* ^*fl/fl*^) were isolated as described^29^. Briefly, kidneys were dissected, minced and digested for 45–60 minutes in a solution of Collagenase A (1 mg/ml) (Roche) and DNAse I. The resulting suspension was strained through a 100-μm strainer and washed 3 times with HBSS buffer. Then the suspension was re-suspended in 30 ml of a 45% Percoll solution (GE-Healthcare BioSciences) in isotonic buffer and centrifuged at 10,000 g for 60 minutes at 4 °C. Glomeruli were enriched in the top band after centrifugation and this band was collected. Cells were washed 3 times with HBSS to remove the Percoll solution. The pellet was resuspended and plated on collagen type I-coated dishes in RPMI 1640 medium with 9% FBS, 100 U/ml penicillin, 100 μg/ml streptomycin, 100 mM HEPES, 1 mM sodium bicarbonate and 1 mM sodium pyruvate. Subculture of primary podocytes was performed by detaching the glomerular cells with 0.25% trypsin-EDTA (Invitrogen), followed by sieving through a 40-μm cell strainer (Falcon; BD Biosciences), and culture on collagen type I–coated dishes. Cells were used at passage 1 for all experiments. For cell treatments LPS was used at a concentration of 10 μg/ml and dexamethasone (DEX) was used at a concentration of 100 nM.

### Immunofluorescence staining of primary podocytes

Cultured isolated primary podocytes were fixed in 4% paraformaldehyde in 1X PBS, permeabilized with 0.1% Triton X-100 in 1X PBS for 10 minutes, blocked with 5% BSA, then incubated with the appropriate antibodies overnight at 4 °C followed by incubation with Alexa Fluor 594–conjugated secondary antibody and Alexa Fluor 488–conjugated phalloidin antibody at RT for 1 hour in the dark. They were washed three times with 1X PBS; then coverslips were mounted with Vectashield. Images were acquired with a Nikon Eclipse Ti laser scanning confocal microscope with a CSU-W1 camera (Andor Technology) using a × 63 Plan Apo (NA = 1.4) oil immersion objective for immunofluorescence analysis, and image analysis and quantitation were performed using NIS elements imaging software and NIH ImageJ software.

### Protamine sulfate perfusion-induced foot process effacement

Perfusion with protamine sulfate (PS) was carried out as previously described^29, 58^. Mice were anesthetized with 1% ketamine and 0.1% xylazine (0.1 ml/10 g body weight) and placed on a heating pad apparatus maintained at 37 °C during the entire experiment. Both kidneys per animal were perfused *in situ* through the left ventricle at a pressure of approximately 120 mmHg and an infusion rate of 9 ml/min. First, both kidneys were perfused with HBSS for 2 minutes, followed by perfusion with HBSS (control group) or PS (2 mg/ml in HBSS, Sigma Aldrich) for 15 minutes. For the heparin treatment group, the kidneys were perfused with heparin (800 μg/ml in HBSS, Santa Cruz biotechnology) for 15 minutes after perfusion with PS for 15 minutes.

At the end of the experiment, kidneys were fixed by perfusion with 2% glutaraldehyde/4% paraformaldehyde in PBS, removed and immersed in the same fixative for transmission electron microscopy (TEM). The degree of foot process effacement was assessed in images by 2 independent observers in a blinded fashion. Image J NIH software was used to measure the foot process width from at least 50 μm GBM per mouse.

### Wound healing assay

Primary podocytes from control and pGR KO mice were isolated, plated and grown to confluence. Then, using a pipet tip, plates were scratched creating a central wound. Cells were allowed to heal for 16 hours before being imaged. Percentage of wound healing was quantified by ImageJ as follows: [(Area T_16 hours_ − Area T_0_)/Area T_0_^27^.

### Western blot

Cells were lysed on ice with lysis buffer (50 mM Tris-HCl pH 7.4, 0.1 mM EDTA, 0.1 mM EGTA, 1% NP-40, 0.1% sodium deoxycholate, 0.1% sodium dodecyl sulfate (SDS), 100 mM NaCl, 10 mM NaF, 1 mM sodium pyrophosphate, 1 mM sodium orthovanadate, 1 mM Pefabloc SC, and 2 mg/ml protease inhibitor cocktail (Roche Diagnostics)). Protein concentrations were determined with the DC Protein assay kit (Bio-Rad Laboratories). Lysates were analyzed by SDS-polyacrylamide gel electrophoresis (PAGE) and immunoblotting. Secondary antibodies were fluorescence-labeled antibodies (LI-COR Biotechnology). Bands were visualized with the Odyssey Infrared Licor system (LI-COR Biotechnology).

### Quantitative PCR

Total RNA was isolated using standard Trizol protocol. RNA was reverse transcribed using the iScript cDNA Synthesis kit (Bio-Rad) and qPCR was performed on a Bio-Rad C1000 Touch thermal cycler using the resultant cDNA, qPCR Master mix and gene specific primers. The following primers were used:

IL-6: 5′ TCTGAAGGACTCTGGCTTTG 3′ and

5′ GATGGATGCTACCAAACTGGA 3′

F3: 5′ CCTTCACGATCTCGTCTGTG 3′ and 5′ CAACCCAAACCCACCAACTA 3′

Nephrin: 5′ GTCGTAGATTCCCCTTGGGT 3′ and

5′ GAGAGTCTATGGCCCACCTG 3′

Synaptopodin: 5′ AGAAGCTACAGTTCTGTTCCCGCA 3′ and

5′ TTCTACAAGAGGCACAAGGCAGGA 3′

Alpha-actinin 4: 5′ ACTACCACGCAGCGAACC-3′ and 5′ TCCCCTGAAATGACCTCC 3′

CD2AP: 5′ TCAGCCACATCCACAAACCAAAGC 3′ and

5′ATTGTTCAGGGTTCCACTCCACCA 3′

RNA isolated from serum was used for IL-6 and F3. RNA from whole kidney was used for nephrin, synaptopodin, alpha-actinin 4 and CD2AP. Gene expression was normalized to the housekeeping gene 18s and is presented as fold change.

### Data availability

No datasets were generated or analyzed during this study.

### Statistical Analyses

Data are expressed as mean ± SEM. Statistical significance was accepted for p < 0.05. Baseline serum and urine measurements were analyzed by t-test. Serum and urine measurements obtained after treatments as well as F-actin data, cell migration data, foot process measurements and Western blot densitometry were analyzed by ANOVA with Tukey’s post-test.

## Electronic supplementary material

Supplementary Information
