# Supplementary material for: Loss of the podocyte glucocorticoid receptor exacerbates proteinuria after injury
Source: Sci Rep. 2017 Aug 29;7:9833. doi: 10.1038/s41598-017-10490-z (PMC5575043; doi:10.1038/s41598-017-10490-z)
Supplement: Supplementary file 1 — Supplementary Information [file 41598_2017_10490_MOESM1_ESM.pdf]

Loss of the podocyte glucocorticoid receptor exacerbates proteinuria after injury

Han Zhou[1#], Xuefei Tian[2#], Alda Tufro[1], Gilbert Moeckel[3], Shuta Ishibe[2], Julie Goodwin[1*]

1 Department of Pediatrics, Yale University School of Medicine, New Haven, CT 06520
2 Department of Internal Medicine, Yale University School of Medicine, New Haven, CT 06520
3 Department of Pathology, Yale University School of Medicine, New Haven, CT 06520

# These authors contributed equally to this work.

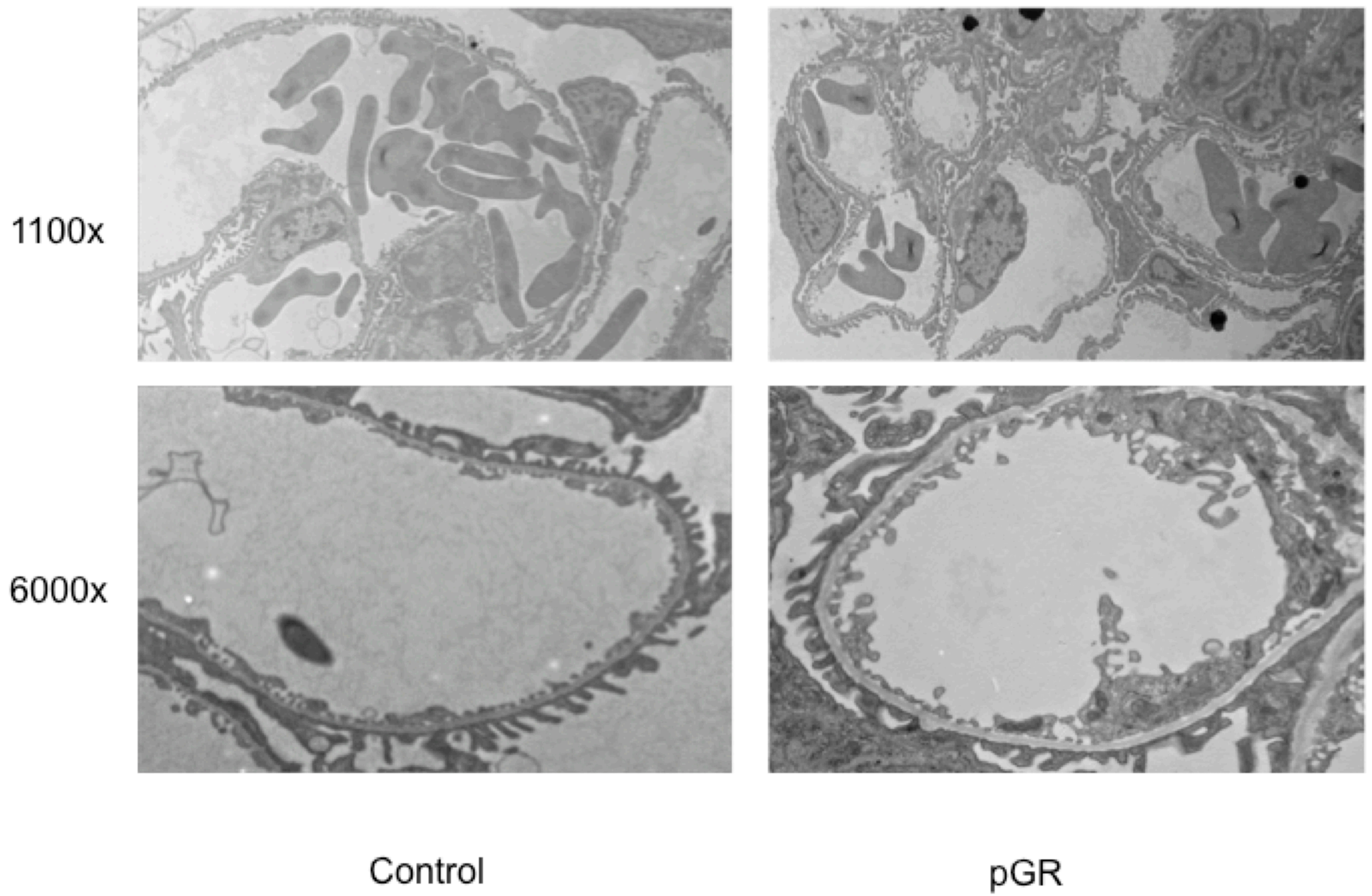

1100x
6000x
Control
pGR

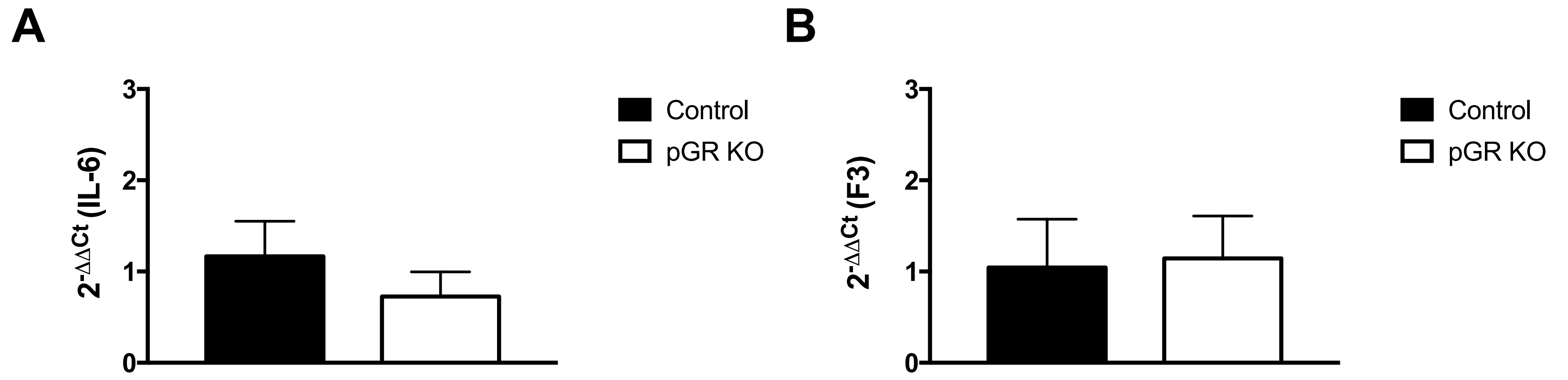
A
B
3
2
1
0
2-ΔΔCt (IL-6)
Control
pGR KO
3
2
1
0
2-ΔΔCt (F3)
Control
pGR KO

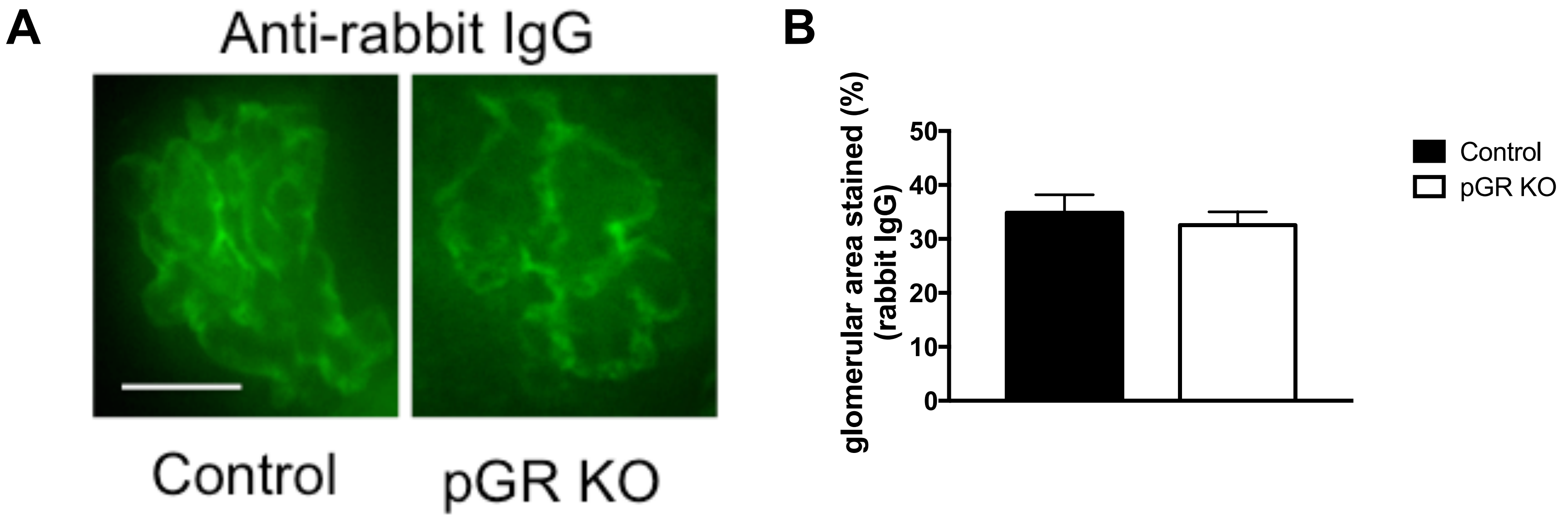
A
Anti-rabbit IgG
Control
pGR KO
B
glomerular area stained (%)
(rabbit IgG)
50
40
30
20
10
0
Control
pGR KO

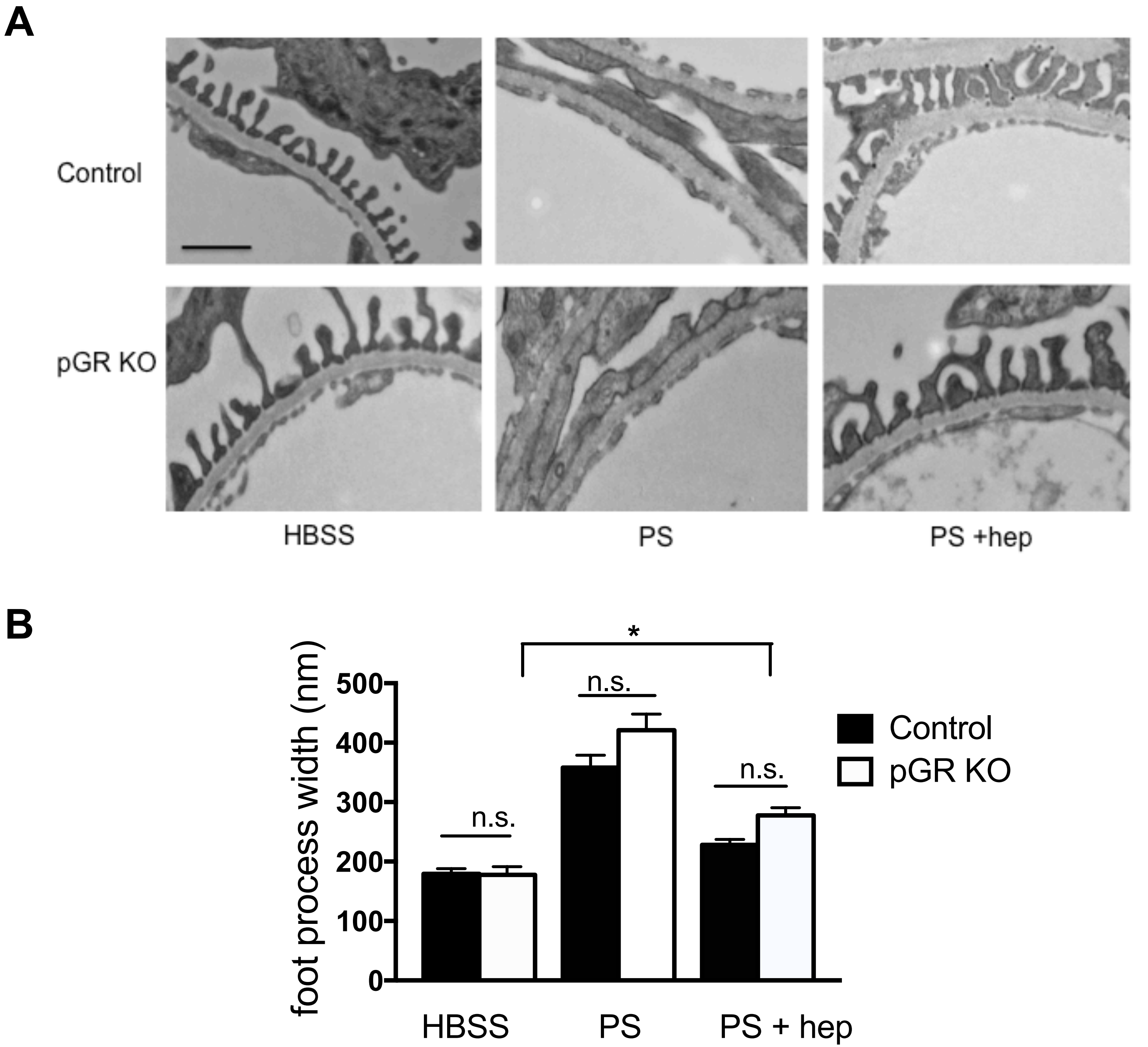
A
Control
pGR KO
HBSS
PS
PS +hep
B
*
n.s.
n.s.
n.s.
500
400
300
200
100
0
foot process width (nm)
Control
pGR KO
HBSS
PS
PS + hep

Supplementary Figure Legends

Figure S1.
Representative TEM images from control and pGR KO mice treated with LPS shown at lower power (1100x) and higher power (6000x). Note the overall worsened foot process effacement in multiple capillary loops that can be seen at lower power in the pGR KO condition.

Figure S2.
Serum was collected from control and pGR KO mice 24 hours after treatment with LPS. RNA was extracted and qPCR for (A) IL-6 and (B) F3 was performed. n=5/group.

Figure S3.
(A) Anti-rabbit IgG antibody staining demonstrates highly localized glomerular basement membrane staining.  Scale bar 50 μm. (B) Quantification of rabbit IgG expression in mice of both genotypes.

Figure S4.
Control and pGR KO mice (n=5/group) were treated with HBSS only, protamine sulfate (PS) only or protamine sulfate and heparin (hep). (A) Representative images of foot process morphology by TEM. Scale bar 1 μm. (B) Quantification of foot process width in each condition.
